# Sustainability and international chemistry collaboration

**DOI:** 10.1093/nsr/nwab037

**Published:** 2021-03-23

**Authors:** Tom Welton

**Affiliations:** Department of Chemistry, Imperial College London, UK; Royal Society of Chemistry

It is my honour to have been invited to contribute, on behalf of the Royal Society of Chemistry (RSC), to this important topical issue on *International Chemistry for a Sustainable Society*. Globally, there are urgent and shared problems we need to solve to create a truly sustainable world, for everyone and for future generations. Chemistry will play a major role in achieving sustainability, whether that is in tackling air pollution in our cities, plastics in our oceans or creating opportunities for economic prosperity.

The international will to address these challenges is growing apace, for example with China and other major economies such as the European Union setting Net Zero targets in advance of the UN Climate Change Conference (COP-26) in the UK later this year. We also see significant commitments by multinational companies to the sustainability agenda, not least in chemical and chemistry-using industries.

It is clear that chemistry will play a major role in achieving the Sustainable Development Goals (SDGs). Within each of the Goals, there are opportunities for chemistry, from new batteries or solar energy conversion technologies to new ways of reducing or recycling waste. By harnessing the collective knowledge, skill and talent across our global chemistry community we will go further and faster in delivering these chemistry solutions. This is why international chemistry collaboration is crucial. Put simply: together we will contribute more.

Collaboration has always been an essential part of my own scientific research. As Professor of Sustainable Chemistry at Imperial College London, I have dedicated my career to sustainability. I cannot imagine being able to do my research without deep collaboration and exchange with international colleagues in both academia and industry.

Now, as President of the RSC, I am in turn proud of our international collaboration. In particular I deeply value our links with sister societies around the world as we work together to champion the international chemical sciences community and to connect scientists across borders as they work to deliver sustainable solutions.

It has been our honour and pleasure to partner with the Chinese Chemical Society (CCS) for many years in this shared endeavour, signing our first formal partnership agreement in 2006. The purpose of our agreement is ‘to co-operate for the establishment of continuous communication and exchange in the field of chemistry and related areas of knowledge for the benefit of the Chinese and UK chemical communities’.

Our China-UK symposia on *Supramolecular Chemistry (2010)* and *Organic Materials for Light Harvesting (2012)* brought together scientists from the UK and China to discuss diverse aspects of how chemistry can contribute towards a sustainable future. Through such joint symposia we have created opportunities for scientists at different career stages to visit and spend time together in universities in both countries. In addition to sharing and advancing knowledge, these visits are important opportunities to build scientific relationships and networks, often leading to future collaboration.

The CCS is also a lead partner in the Chemical Science and Society (CS3) summit, which convenes scientists and funding bodies from the partner countries to discuss how chemistry can contribute to addressing global challenges. For example, in 2017, the summit in Dalian focused on *Solar Energy and Photonics for a Sustainable Future*, while in 2019 in London the theme was *Science to Enable Sustainable Plastics*.

China has been clear in its commitment to sustainability, with the concept of ecological civilization written into its very constitution. Its 14th Five Year Plan has set out ambitious goals for green and low-carbon development, while its scientists are leading the way right across the spectrum of sustainability research. This is reflected in the deep and wide-ranging expertise of RSC Fellows in China and some of our recent China Fellows Forums, which have focused on topics such as water safety and clean energy.

While we cannot forecast the future, I believe that we can be confident that by working together across borders we will always be in a much stronger position to address global challenges, whatever they might be. Our partnership with the CCS will therefore only grow in importance, as we work to strengthen scientific collaboration between our two countries and across the global chemical sciences community.

***Conflict of interest statement*.** None declared.

